# Step-freeze-drying method for carbon aerogels: a study of the effects on microstructure and mechanical property

**DOI:** 10.1039/c9ra01328h

**Published:** 2019-03-29

**Authors:** Lei Hu, Rujie He, Zhang Lu, Keqiang Zhang, Xuejian Bai

**Affiliations:** Institute of Advanced Structure Technology, Beijing Institute of Technology Beijing 100081 China herujie@bit.edu.cn; Beijing Key Laboratory of Lightweight Multi-functional Composite Materials and Structures, Beijing Institute of Technology Beijing 100081 China

## Abstract

In this paper, a novel step-freeze-drying method was used to prepare carbon aerogels. The effects of step-freeze-drying on the density, linear shrinkage, specific surface area, pore size distribution, microstructure and compressive strength of carbon aerogels were investigated, and compared to traditional freeze-drying methods. It was found that the step-freeze-drying method reduced the density, linear shrinkage and pore size of carbon aerogels compared to traditional freeze-drying. And it also improved the specific surface area, the microstructural homogenization and the compressive strength of carbon aerogels compared to traditional freeze-drying. It is therefore believed that step-freeze-drying is an efficient method to obtain carbon aerogels with fine microstructure and high mechanical property.

## Abstract 2

In this paper, a novel step-freeze-drying method was used to prepare carbon aerogels.

## Graphical abstract

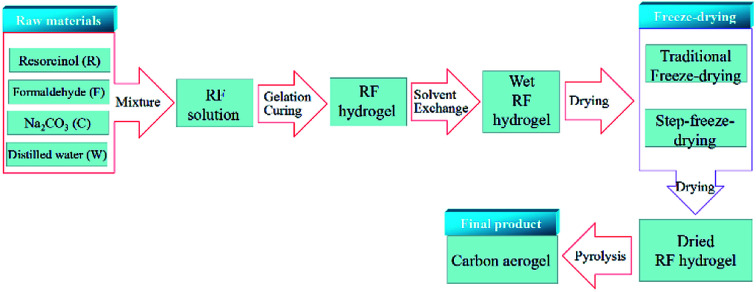

## Introduction

Carbon aerogel is a kind of novel nano-scaled carbonaceous material,^1,2^ which has attracted extensive interest due to its specific properties, such as high porosity, low density, high surface area, large pore volume and good thermal stability.^3–5^ These properties mean carbon aerogels show good application prospects in thermal, acoustic, electrical and catalytic fields. Carbon aerogel is expected to be used as an electric double layer capacitor, adsorbent, catalyst support, and material for high-temperature thermal insulators.^6–9^

Carbon aerogel is usually obtained from the pyrolysis of an organic precursor.^10,11^ There are always four typical stages during the preparation of carbon aerogels, including gelation and aging of the sol mixture, solvent exchange, drying of the wet gel, and carbonization of the dried gel.^12,13^ Many reports have focused on the effects of each step on the green/final microstructure and properties of the carbon aerogel.^14–16^ Especially, it is found that drying process of the wet gel is one of the most important processes during the fabrication of carbon aerogels.^17^

Usually three typical drying methods, including ambient pressure drying,^18^ supercritical drying^19^ and freeze-drying,^20^ were used to dry the wet gel. Unfortunately, ambient pressure drying always cause significant shrinkage of the aerogel, and supercritical drying usually is of very high cost. Therefore, freeze-drying, a more economical and efficient method, is used to obtain carbon aerogel with lower linear shrinkage, homogeneous microstructure, high mechanical property and low cost. Yamamoto *et al.*^21,22^ reported the synthesis of carbon aerogels by using freeze-drying method, and found the as-obtained mesoporous carbon aerogels had high surface areas and large mesopore volumes than other drying methods. However, the freeze-drying conditions and protocols always had significant influences on the properties of carbon aerogels.^22^ Interesting, in our study, it was found that conducting the freeze-drying step by step, named as step-freeze-drying method, could effectively improve the properties of as-prepared carbon aerogels comparing to traditional freeze-drying method, especially the microstructure and mechanical properties.

In this paper, carbon aerogels were produced by using a step-freeze-drying method and traditional freeze-drying method. The effects of step-freeze-drying on the density, linear shrinkage, specific surface area, pore size distribution, microstructure and compressive strength of carbon aerogels were investigated. The applicability and advantage of step-freeze-drying were discussed and compared with traditional freeze-drying. It is believed that this study can give some methods to obtain carbon aerogels with fine microstructure and high mechanical property.

## Experimental

### Raw materials

Carbon aerogels were obtained by the pyrolysis of RF cryogels in this work. RF cryogels were synthesized by the polycondensation of resorcinol (R) and formaldehyde (F), using sodium carbonate (Na_2_CO_3_, C) as the basic catalyst, and distilled water (W) as the diluent. *t*-Butanol were as the medium for step-freeze-drying and traditional freeze-drying. All the raw materials were chemically pure and purchased from Beijing Tong Guang Fine Chemicals Co., Ltd., China.

### Fabrication

**Fig. 1:**
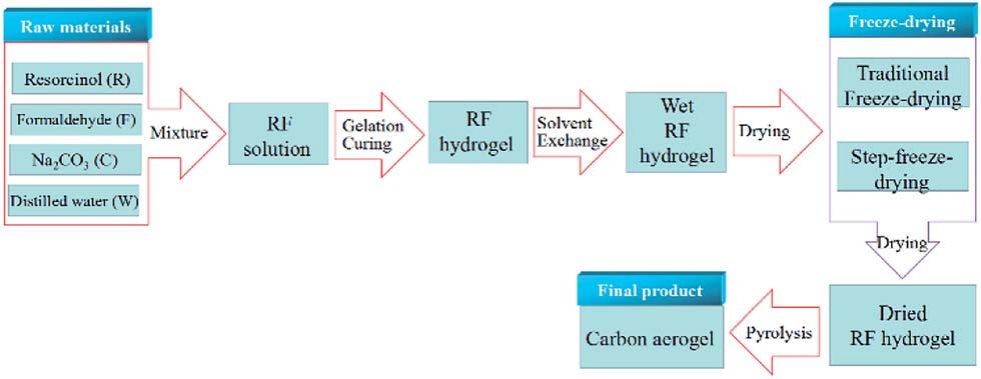
The flow chart of the preparation of carbon aerogels.

The fabrication procedure of carbon aerogels was consisted of three main steps, as shown in Fig. 1.

#### (1) Synthesis of RF hydrogel

RF hydrogels were synthesized by sol–gel polycondensation of resorcinol (R) with formaldehyde (F) according to the method proposed by Pekala *et al.*^23^ Here, sodium carbonate (NaCO_3_, C) was used as a basic catalyst. Firstly, RF solutions were prepared from the mixture of resorcinol, formaldehyde, sodium carbonate and distilled water. The mole ratio of formaldehyde to resorcinol (F/R, mol mol^−1^) was set as 0.5, the mole ratio of resorcinol to catalyst (R/C, mol mol^−1^) ranged from 200 to 600, and the ratio of water to resorcinol (W/R, mol mol^−1^) was chosen as 100. Then, the RF solutions were poured into glass tubes and gelled by curing at 323 K for 1 day and subsequently at 358 K for 2 days. After that, RF hydrogels were finally obtained.

#### (2) Drying of RF hydrogels

To replace the water filling in the RF hydrogels, *t*-butanol was reported to be effective drying solvent to remove the water with retaining the pores and shortening the freeze-drying time simultaneously.^24^ In this work, RF hydrogels were successively immersed into *t*-butanol aqueous medium (mole concentrations were 25, 50, 75 and 100%, respectively).

**Fig. 2:**
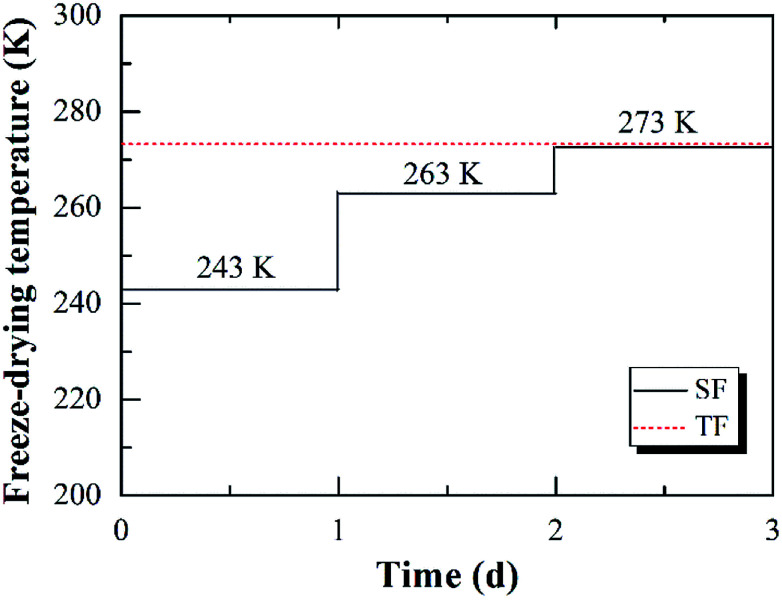
The comparison of step-freeze-drying (SF) and traditional freeze-drying (TF).

The wet RF hydrogels were freeze-dried step by step. The wet RF hydrogels were firstly dried at 243 K for 1 day, then at 263 K for 1 day, and finally at 273 K for 1 day, using a vacuum freeze-drying equipment (LGJ-10, Songyuan Freeze Dryer Co., Ltd., China). Dried RF hydrogels were finally obtained. This novel drying method was named as step-freeze-drying (SF for simplicity). For comparison, the wet RF hydrogels were also dried directly at 273 K for 3 days. This drying method was named as traditional freeze-drying (TF for simplicity). The difference between SF and TF was presented in Fig. 2.

#### (3) Pyrolysis of carbon aerogels

The dried RF hydrogels were pyrolyzed to obtain carbon aerogels. The carbon aerogels were prepared in a conventional graphite furnace (ZT-70-23Y, Shanghai Chenxin Furnace Co., Ltd., China). Before pyrolysis, N_2_ gas was flowed to replace the air inside the furnace. The furnace was firstly heated to 1073 K at a heating rate of 1 K min^−1^ and then maintained at 1073 K for 1 h. After pyrolysis, the furnace was cooled to room temperature naturally.

### Characterization

The bulk density (*ρ*) of the dried RF hydrogel and pyrolyzed carbon aerogel was obtained by measuring the volume and the weight. The linear shrinkage of the dried RF hydrogel and pyrolyzed carbon aerogel was calculated from the dimensions before and after drying and pyrolysis. Nitrogen adsorption measurements were performed to obtain the pore properties, such as specific surface area and specific pore volume, by using a BELSORP-max analyser (Microtracbel, Japan). The desorption branches were used for the Barrett–Joyner–Halenda (BJH) calculation. The microstructure of the pyrolyzed carbon aerogel was investigated by using a JSM-6700F scanning electron microscope (SEM) (JEOL, Japan) after coating the samples with a thin platinum layer. Compressive strength of the pyrolyzed carbon aerogel was measured using a compressive testing machine (LEGEND 2367, INSTRON Co., USA) on *∅*10 mm × 20 mm (diameter × height) testing bars with a crosshead speed of 0.05 mm min^−1^ at room temperature. For each testing conditions, at least three specimens were used to obtain the average value.

## Results and discussion

### Density and linear shrinkage

**Fig. 3:**
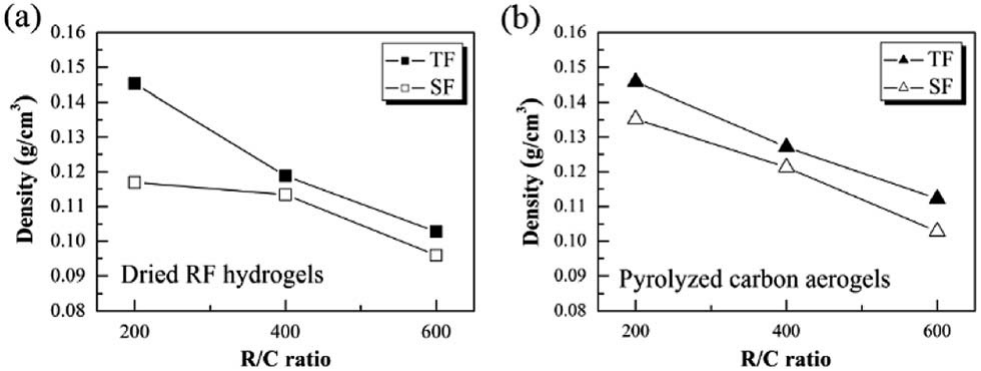
Effect of step-freeze-drying on the density of (a) the dried RF hydrogels and (b) the pyrolyzed carbon aerogels.

Fig. 3a and b show the density of the as-prepared dried RF hydrogels and the pyrolyzed carbon aerogels with different R/C ratios by using the novel step-freeze-drying method (SF) and traditional freeze-drying method (TF), respectively. For traditional freeze-drying method, the density of the dried RF hydrogels with an R/C ratio of 200, 400 and 600 was 0.145, 0.118 and 0.102 g cm^−3^, respectively. Whereas for step-freeze-drying method, the density of dried RF hydrogels was much smaller. The density of dried RF hydrogels with an R/C ratio of 200, 400 and 600 was reduced to 0.117, 0.113 and 0.096 g cm^−3^, respectively (as shown in Fig. 3a). Moreover, for traditional freeze-drying method, the density of the pyrolyzed carbon aerogels with an R/C ratio of 200, 400 and 600 was 0.145, 0.127 and 0.112 g cm^−3^, respectively. However, for step-freeze-drying method, the density of the pyrolyzed carbon aerogels with an R/C ratio of 200, 400 and 600 was reduced to 0.135, 0.121 and 0.103 g cm^−3^, respectively (as shown in Fig. 3b). Obviously, compared to traditional freeze-drying, both the dried RF hydrogels and the pyrolyzed carbon aerogels by step-freeze-drying had lower density. Besides, it was also found that, as the R/C radios increasing, the density decreased correspondingly for both the dried RF hydrogels and the pyrolyzed carbon aerogels no matter what types of drying method used. What's more, the density of both the dried RF hydrogels and the pyrolyzed carbon aerogels made by step-freeze-drying method were smaller than those made by traditional freeze-drying method. The differences might be because both the drying and pyrolysis process were more complete for the step-freeze-drying method. It was thus known that step-freeze-drying was an efficient method to further reduce for both the dried RF hydrogels and the pyrolyzed carbon aerogels.

**Fig. 4:**
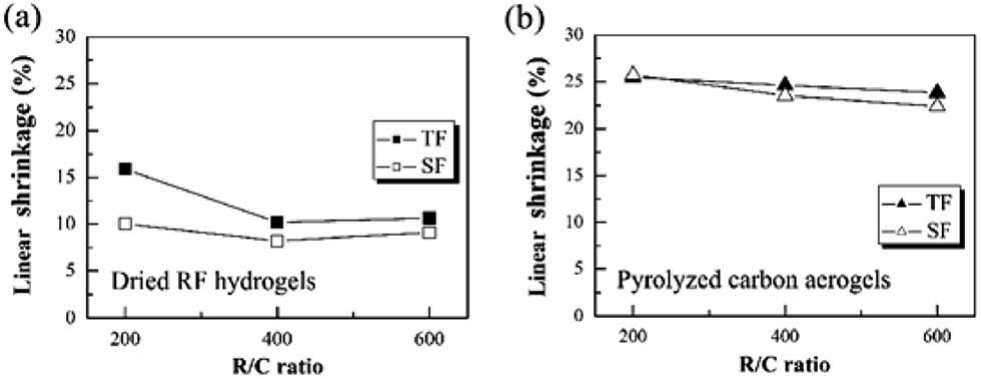
Effect of step-freeze-drying on the linear shrinkage of (a) the dried RF hydrogels and (b) the pyrolyzed carbon aerogels.

The effect of step-freeze-drying on the linear shrinkage of the dried RF hydrogels and the pyrolyzed carbon aerogels are given in Fig. 4. Both the linear shrinkage during the drying process and pyrolysis process were investigated by changing the R/C ratios. It could be found that, the linear shrinkage decreased with increasing the R/C ratio for both traditional freeze-drying and step-freeze-drying method. The reason might be that less capillary tensions resulted from the wider pore size, which was formed in a lower catalyst concentration (higher R/C ratio). For traditional freeze-drying method, the linear shrinkage of dried RF hydrogels with an R/C ratio of 200, 400 and 600 was 15.89, 10.19 and 10.64%, respectively. Whereas for step-freeze-drying method, the linear shrinkage of dried RF hydrogels with a R/C ratio of 200, 400 and 600 was reduced to as low as 10.03, 8.18 and 9.11%, respectively (as shown in Fig. 4a). Besides, for traditional freeze-drying method, the density of pyrolyzed carbon aerogels with an R/C ratio of 200, 400 and 600 was 25.48, 24.61 and 23.83%, respectively. However, for step-freeze-drying method, the density of pyrolyzed carbon aerogels with an R/C ratio of 200, 400 and 600 was reduced to 25.73, 23.53 and 22.41%, respectively (as shown in Fig. 4b). Meanwhile, the linear shrinkage during both the step-freeze-drying and subsequent pyrolysis was much less than those for traditional freeze-drying. We calculated the weight loss rate of gel drying after two drying methods, and found that there was no significant difference between them. This shows that the two drying methods have basically achieved complete drying, but the linear shrinkage in the drying process is significantly different. This indicates that the change of the two RF hydrogels densities after different drying methods is mainly caused by different linear shrinkage rates during drying process. Thus we found that the step-freeze-drying made carbon aerogels changed less during the drying process and the pyrolysis process.

### Specific surface area and pore size distribution

**Fig. 5:**
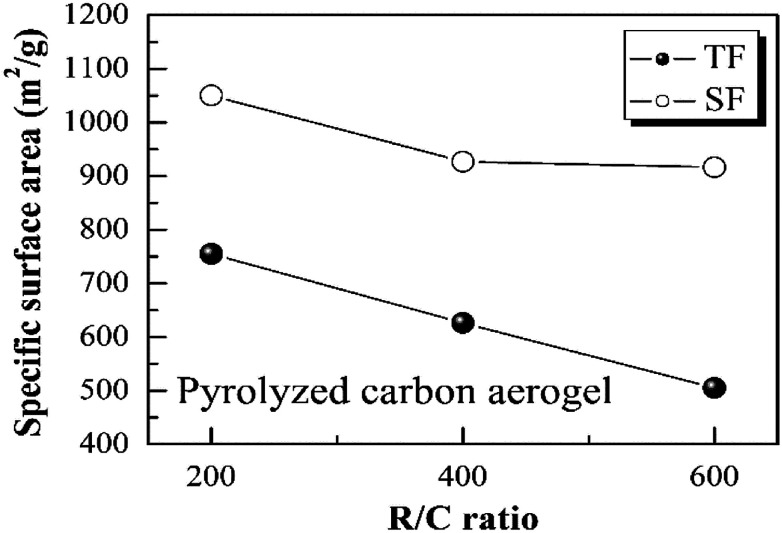
Effect of step-freeze-drying on the specific surface area of carbon aerogels.

Importantly, the step-freeze-drying process not only affected the density of the pyrolyzed carbon aerogels macroscopically, but also had a deeper effect on their pore structure, specific surface area and pore size distribution. Fig. 5 shows the Brunauer–Emmett–Teller (BET) specific surface area of the pyrolyzed carbon aerogels prepared from different R/C ratios by different drying methods. It was observed that all the pyrolyzed carbon aerogels with different R/C ratios had high surface areas above 500 m^2^ g^−1^. For traditional freeze-drying method, the specific surface area of the pyrolyzed carbon aerogels with an R/C ratio of 200, 400 and 600 was 754.85, 626.26 and 505.05 m^2^ g^−1^, respectively. When using the step-freeze-drying method, the specific surface area of the pyrolyzed carbon aerogels was significantly improved. The specific surface area of the pyrolyzed carbon aerogels with an R/C ratio of 200, 400 and 600 was as high as 1050.10, 926.76 and 916.55 m^2^ g^−1^, respectively. Obviously, the specific surface area of carbon aerogels made by step-freeze-drying method was higher than that by traditional freeze-drying method. These results further indicated that step-freeze-drying method was favour for producing carbon aerogels with small pore size.

**Fig. 6:**
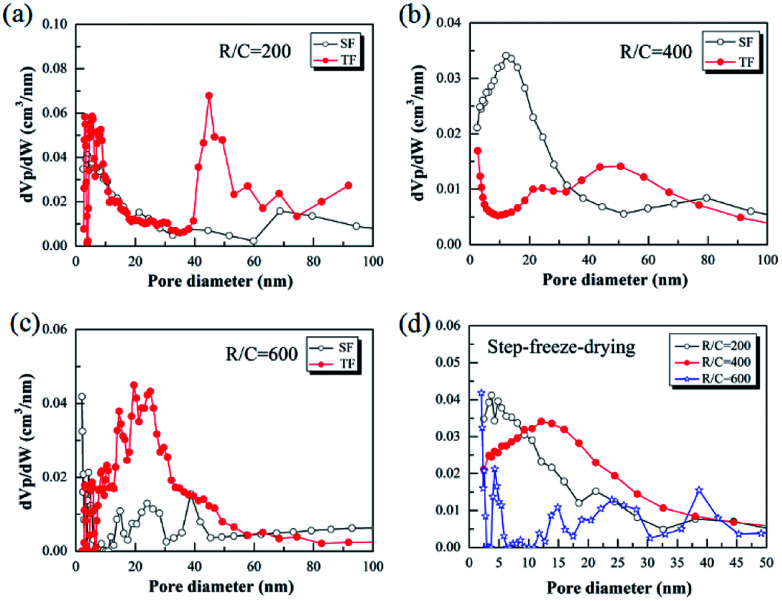
Effect of step-freeze-drying on the pore size distribution of the pyrolyzed carbon aerogels: (a) R/C = 200; (b) R/C = 400; (c) R/C = 600; (d) comparison.

Fig. 6 shows the Barrett–Joyner–Halenda (BJH) pore size distributions of the pyrolyzed carbon aerogels prepared by using step-freeze-drying and traditional freeze-drying, respectively. It could be found that the pyrolyzed carbon aerogels had sharp pore size distribution and were mostly mesoporous. For a same R/W ratio, the peak pore diameter increased with the increasing of R/C ratio. By comparing the pore size distribution of the pyrolyzed carbon aerogels prepared by step-freeze-drying and traditional freeze-drying, the peak pore diameter of the carbon aerogels fabricated by step-freeze-drying was smaller than that by traditional freeze-drying, indicating that the pores of carbon aerogels produced by step-freeze-drying were smaller than those by traditional freeze-drying. This phenomena was due to the destruction of the structure and fast sublimation of frozen solvent which would lead to the growth of pores during the freeze-drying process. Typically, the step-freeze-drying method improved the situation significantly.

Table 1 is the test data of carbon aerogels density and specific surface area in the public literature. By comparing the data with other literatures, we can see from the table that the step-freeze-drying method adopted in this experiment effectively reduced the density of the prepared carbon aerogels and greatly increased the specific surface area of the carbon aerogels comparing with other drying methods and traditional freeze-drying.

**Table 1:** Comparison of the as-obtained carbon aerogels with reported data in the open literature

Authors	R/C	Dry	Density (g cm^−3^)	*S*_BET_ (m^2^ g^−1^)
Bock^25^	200	Supercritical drying	0.381	—
Tamon^20^	25	Freeze-drying	0.32	503
Yamamoto^21^	200	Freeze-drying	—	427–552
Wiener^9^	—	Ambient drying	∼0.3	133
Feng^26^	357	Supercritical drying	0.052	666
This work	600	Freeze-drying	0.112	505
This work	600	Step-freeze-drying	0.103	916

### Microstructure and compressive strength

**Fig. 7:**
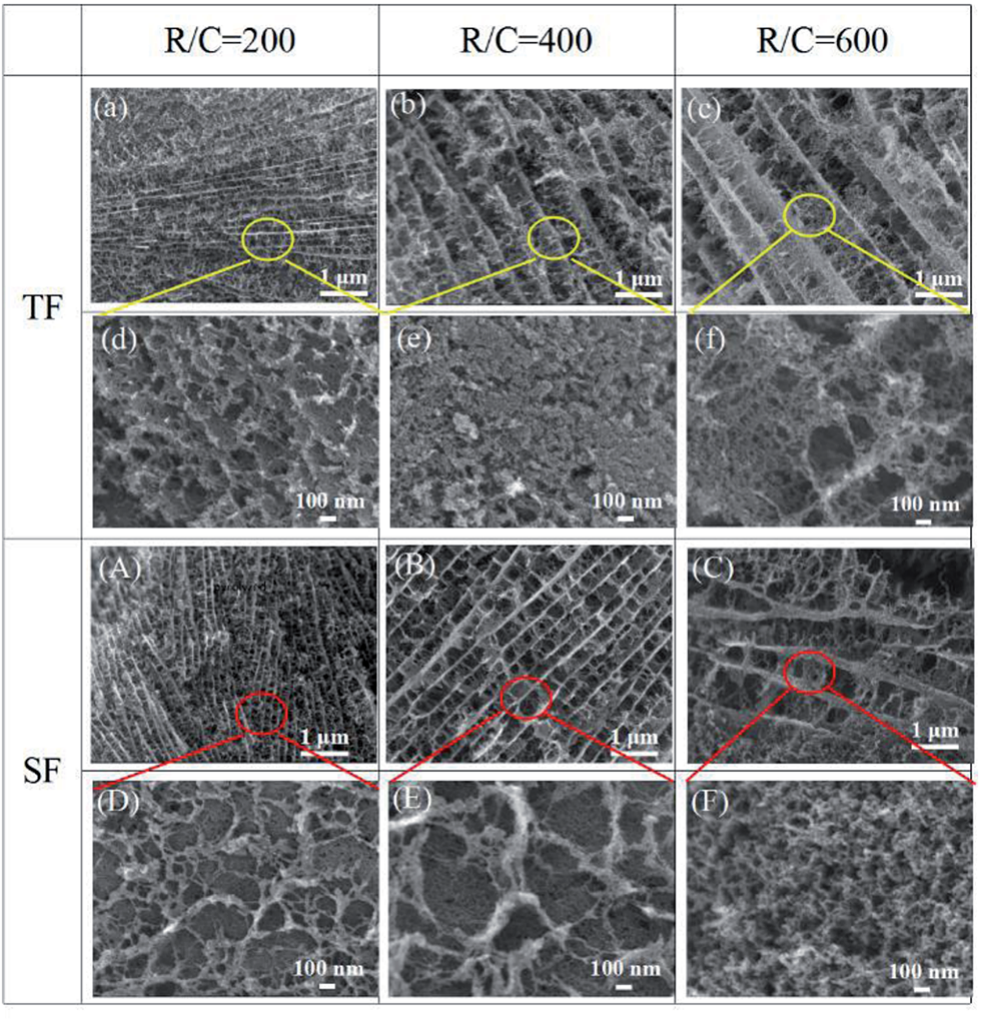
Effect of step-freeze-drying on the microstructure of the carbon aerogel: TF: (a and d) R/C = 200; (b and e) R/C = 400; (c and f) R/C = 600. SF: (A and D) R/C = 200; (B and E) R/C = 400; (C and F) R/C = 600.

Fig. 7 shows the scanning electron micrographs of the final pyrolyzed carbon aerogels prepared using traditional freeze-drying and step-freeze-drying. From Fig. 7, it could found that the pyrolyzed carbon aerogels made by traditional freeze-drying had some large cracks on the surface due to the destruction of the structure and fast sublimation of frozen solvent, which would also seriously affect the mechanical properties of the carbon aerogels. Fortunately, it was also found that the microstructure of the pyrolyzed carbon aerogels made by step-freeze-drying were more homogeneous and less-cracks. For aerogel, the aerogel particles would form multi-layered layered structures, and the layers were connected by aggregated particles. For carbon aerogels produced in this work, the distance between these layers ranged from 0.1 to 1 μm, and the particle sizes ranged from 10 to 30 nm. By comparing the microstructure of the carbon aerogels prepared by step-freeze-drying and traditional freeze-drying, it was found that the distance between the layers of carbon aerogels made by traditional freeze-drying was much larger, their microstructure was more loose and their pore size was much larger than that of carbon aerogels made by step-freeze-drying, but the size of the skeleton particles forming aerogels had no obvious change. These results further indicated that step-freeze-drying technology was helpful for obtaining carbon aerogels with much smaller and finer microstructure.

**Fig. 8:**
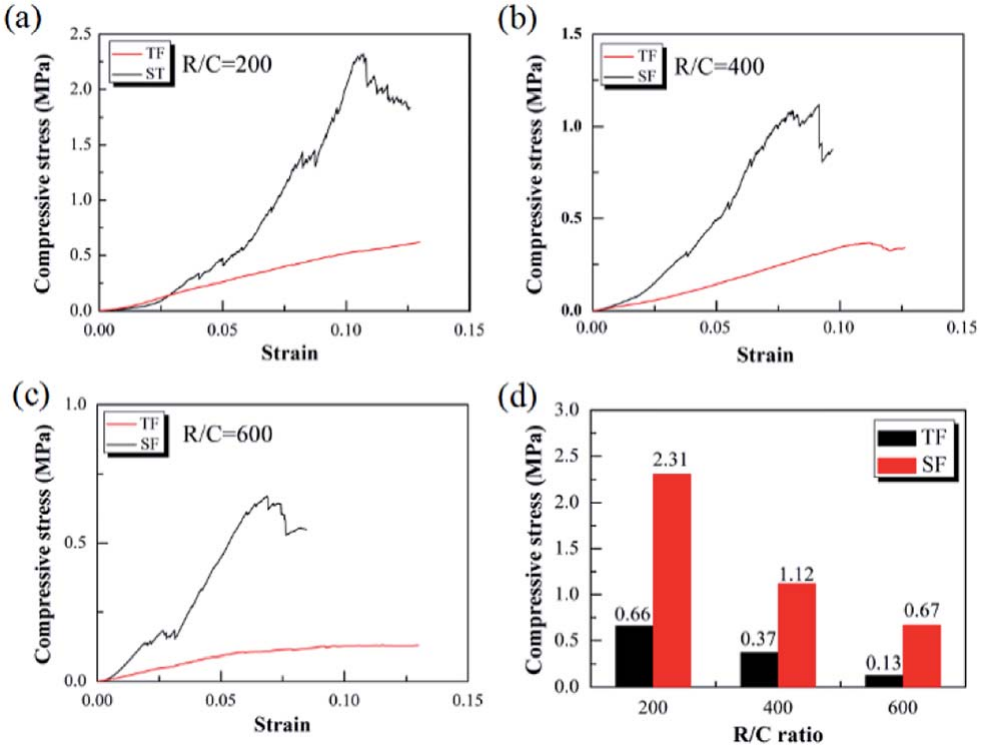
Effects of step-freeze-drying on the compressive strength of the carbon aerogel: (a) R/C = 200; (b) R/C = 400; (c) R/C = 600; (d) comparison.

Fig. 8 presents the compressive stress–strain curves of the pyrolyzed carbon aerogels prepared by step-freeze-drying and traditional freeze-drying, respectively. It was observed that, the compressive stress increased proportionally to the strain firstly and abruptly declined at the highest point when the specimen was broken, which indicated that the carbon aerogels were still very brittle. When the R/C ratio was 200, the compressive strength of carbon aerogels by step-freeze-drying and traditional freeze-drying was 2.31 and 0.66 MPa, respectively. When the R/C ratio was 400, the compressive strength of carbon aerogels by step-freeze-drying and traditional freeze-drying was 1.12 and 0.37 MPa, respectively. And when the R/C ratio was 600, the compressive strength of carbon aerogels by step-freeze-drying and traditional freeze-drying was 0.67 and 0.13 MPa, respectively. Obviously, for both carbon aerogels by step-freeze-drying and traditional freeze-drying, their compressive strength decreased with the R/C ratio increasing, which was due to the smaller density and larger pore size with the R/C ratio increasing. Most importantly, it was clearly found that the compressive strength of the carbon aerogels made by step-freeze-drying method was much higher than that by traditional freeze-drying when the R/C value was the same, indicating the step-freeze-drying method could effectively improve the mechanical properties of the aerogel and made them more compressive. This was because step-freeze-drying could maintain the microstructure and framework of carbon aerogels, prevented the shrinkage, cracking and collapse of carbon aerogels during drying process, and improved the final properties of pores and density of aerogels, which ultimately improved the mechanical properties of aerogels and greatly enhanced the future applications of carbon aerogels.

## Conclusions

In this paper, step-freeze-drying method, a novel drying method different from traditional freeze-drying, was used to prepare carbon aerogels. The effects of step-freeze-drying on the density, linear shrinkage, specific surface area, pore size distribution, microstructure and compressive strength on the final carbon aerogels were investigated. Some main conclusions were listed as follows,

(1) RF hydrogels were synthesized by the sol–gel polycondensation of resorcinol with formaldehyde. Dried RF hydrogels could be obtained using step-freeze-drying and traditional freeze-drying methods. And finally carbon aerogels were obtained from the pyrolysis of dried RF hydrogels.

(2) For step-freeze-drying, both the density of the dried RF hydrogels and the pyrolyzed carbon aerogels were smaller than those by traditional freeze-drying. Meanwhile, the linear shrinkage of the dried RF hydrogels and the pyrolyzed carbon aerogels by step-freeze-drying was much less than those by traditional freeze-drying.

(3) Step-freeze-drying method also increased the specific surface area of carbon aerogels and was helpful for obtaining carbon aerogels with much smaller pore size.

(4) The microstructure of the carbon aerogels by step-freeze-drying were finer than those by traditional freeze-drying. Besides, the compressive strength of the carbon aerogels by step-freeze-drying was higher than those by traditional freeze-drying. The reason was that step-freeze-drying could maintain the microstructure and framework of carbon aerogels well, thus effectively improved their mechanical properties.

Therefore, it is believed that step-freeze-drying method can be helpful for obtaining carbon aerogels with fine microstructures and high mechanical properties, and finally promoting their engineering applications.

## Conflicts of interest

There are no conflicts to declare.

## Supplementary Material
